# Longitudinal kinetics of neutralizing antibodies against circulating SARS-CoV-2 variants and estimated level of group immunity of booster-vaccinated individuals during omicron-dominated COVID-19 outbreaks in the Republic of Korea, 2022

**DOI:** 10.1128/spectrum.01655-23

**Published:** 2023-09-26

**Authors:** Young Jae Lee, Ju-yeon Choi, Jinyoung Yang, Jin Yang Baek, Hye-Jin Kim, Su-Hwan Kim, Hyeonji Jeong, Min-Seong Kim, Hye Won Lee, GaRim Kang, Eun Joo Chung, Tae-Yong Kim, Hyo-jeong Hong, Sang Eun Lee, Yeong Gyeong Jang, Sung Soon Kim, Kyong Ran Peck, Jae-Hoon Ko, Byoungguk Kim

**Affiliations:** 1 Division of Vaccine Clinical Research, Center for Vaccine Research, National Institute of Infectious Diseases, Korea National Institute of Health, Cheongju, South Korea; 2 Division of Infectious Diseases, Department of Medicine, Samsung Medical Center, Sungkyunkwan University School of Medicine, Seoul, South Korea; 3 Asia Pacific Foundation for Infectious Diseases (APFID), Seoul, South Korea; Barcelona Centre for International Health Research (CRESIB, Hospital Clínic-Universitat de Barcelona), Barcelona, Spain

**Keywords:** cross reactions, antibodies, neutralizing, kinetics, breakthrough infections, vaccination, SARS-CoV-2 variants

## Abstract

The coronavirus disease 2019 pandemic persisted for 3 years and is now transitioning to endemicity. We illustrated the change in group immunity induced by vaccination (monovalent vaccines) and breakthrough infections (BIs) in a healthcare worker (HCW) cohort. Five sampling points were analyzed: before the third dose and 1, 3, 5, and 8 months after the vaccination. The last two points corresponded roughly to 1 and 4 months after omicron BA.1/BA.2 BI. A semi-quantitative anti-spike binding antibody (Sab) assay and plaque reduction neutralization test (PRNT) against circulating variants were conducted. A linear regression model was utilized to deduce correlation equations. Baseline characteristics and antibody titers after the third dose were not different between 106 HCWs with or without BI (54/52). One month after the third dose, BA.1 PRNT increased with wild-type (WT), but 3 months after the third dose, it decreased more rapidly than WT PRNT. After BI, BA.1 PRNT increased robustly and waned slower than WT. A linear equation of waning kinetics was deduced between log_10_Sab and months, and the slope became gradual after BI. The estimated BA.5 PRNT titers at the beginning of the BA.5 outbreak were significantly higher than the BA.1 PRNT titers of the initial BA.1/BA.2 wave, which might be associated with the smaller size of the BA.5 wave. BA.1/BA.2 BI after the third dose elicited robust and broad neutralizing activity, preferentially maintaining cross-neutralizing longevity against BA.1 and BA.5. The estimated kinetics provide an overview of group immunity through the third vaccination and BA.1/BA.2 BI, correlating with the actual outbreaks.

**IMPORTANCE:**

This study analyzed changes in group immunity induced by coronavirus disease 2019 (COVID-19) vaccination and BA.1/BA.2 breakthrough infections (BIs) in a healthcare worker cohort. We investigated the longitudinal kinetics of neutralizing antibodies against circulating variants and confirmed that BA.1/BA.2 BIs enhance the magnitude and durability of cross-neutralization against BA.1 and BA.5. Correlation equations between semi-quantitative anti-spike antibody and plaque reduction neutralization test titers were deduced from the measured values using a linear regression model. Based on the equations, group immunity was estimated to last up to 11 months following the third dose of the COVID-19 vaccine. The estimated group immunity suggests that the augmented immunity and flattened waning slope through BI could correlate with the overall outbreak size. Our findings could provide a better understanding to establish public health strategies against future endemicity.

## INTRODUCTION

The coronavirus disease 2019 (COVID-19) pandemic has continued over 3 years, with successive waves of outbreaks driven by immune-escaping severe acute respiratory syndrome coronavirus 2 (SARS-CoV-2) variants (1, 2). In the Republic of Korea, the wild-type (WT) SARS-CoV dominated the outbreak waves in 2020, followed by the delta variant in 2021 and the omicron variant in 2022 (2, 3). The omicron variant, designated as the fifth variant of concern (VOC) on 26 November 2021, has remained the sole VOC, and waves of outbreaks dominated by its subvariants continue globally (4, 5). The spread of the omicron variant led to the fifth domestic outbreak wave in the Republic of Korea, which surged in February 2022 and waned in April 2022. The transition of the subvariant from BA.1 to BA.2 occurred in March 2022, recording the largest outbreak since the emergence of COVID-19. This was followed by the sixth domestic outbreak wave caused by BA.5 in 2022. At the end of the fifth wave (18 April 2022), despite the relaxation of social distancing, the following outbreak waves caused by omicron subvariants showed a tendency to decrease in size over time, suggesting the development of herd immunity through vaccination and natural infections.

From February 2022, breakthrough infections (BIs) rapidly increased despite high vaccine coverage due to the waning of vaccine-induced immunity and the immune-evading nature of the omicron variant (6). Hybrid immunity induced by delta or omicron BI is known to elicit cross-reactive neutralization against VOCs, including BA.1 (7 – 10), but insufficient boosting and rapid waning of cross-reactivity are also concerned due to antigenic imprinting (11 – 14). Antigenic imprinting refers to a phenomenon by which the immune system is fixated on the initial exposure to a pioneering strain, limiting antibody responses to different variants (13, 14). The potential imprinting of WT SARS-CoV-2 could undermine variant-specific antibody responses following heterologous BI (11, 12, 14). Herein, we investigated the magnitude and breadth of neutralizing activities against circulating SARS-CoV-2 variants during 2022, including delta and omicron subvariants in addition to WT, waning after a third dose of COVID-19 vaccine and augmented through BI during the BA.1/BA.2 outbreak period in a long-term follow-up of the healthcare worker (HCW) vaccinee cohort (15). Furthermore, the longevity of neutralizing activity and changing group immunity were estimated using the correlation equations deduced from the measured titers of the semi-quantitative anti-spike antibody (Sab) test and plaque reduction neutralizing test (PRNT) against WT SARS-CoV-2, BA.1, and BA.5 subvariants, up to 11 months following the third vaccination of the COVID-19 vaccine (16 – 18).

## MATERIALS AND METHODS

### Study population and specimen collection

As the third-dose vaccination schedule for HCWs varied between participating centers, data from the largest center in a nation-wide multicenter vaccinee cohort were utilized to reflect a relatively homogenous vaccination timing (15). The study population was divided into two groups based on primary vaccination schedules: the BNT-BNT group received two doses of BNT162b2 (BNT; Comirnaty, Pfizer, NY, USA), and the ChAd-BNT group received ChAdOx1 (ChAd; Vaxzevria, AstraZeneca, Oxford, UK), followed by BNT162b2. Those who did not receive a third dose or who experienced a SARS-CoV-2 infection before the third vaccination were excluded. In addition to the individual report about the history of infection, an anti-nucleocapsid antibody (Nab) test was performed to screen those with previous infections from the baseline sample (19). A standard dose of the BNT vaccine was provided as the third dose on 28 October 2021, and most of the included HCWs were vaccinated by December 2021. The occurrence of BI during the BA.1/BA.2 outbreak period was observed between 26 January and 13 July 2022.

Five sampling points were included in the present analysis: 1 or 2 months before the third dose (from September to November 2022, depending on the schedule of the third dose) and 1, 3, 5, and 8 months after the third dose. The last sampling was conducted mostly in July 2022. As BI during the BA.1/BA.2 outbreak period occurred within a relatively narrow time window, the 5- and 8-month sampling points after the third dose correspond roughly to 1 and 4 months after BI for individuals who experienced such an infection. The PRNT result before and 1 month after the third dose was published previously (17). The diagnosis of SARS-CoV-2 infection was primarily based on positive rapid antigen or reverse transcription-polymerase chain reaction (RT-PCR) tests of respiratory specimens (molecular diagnosis). HCWs with either a positive conversion of Nab or a greater than fourfold increase of Sab that was not related to the booster vaccination were also considered to be infected with SARS-CoV-2 (serologic diagnosis) (6). The presumed variant type of SARS-CoV-2 infecting each HCW was based on the dominant strain circulating during the outbreak period. All participants provided written informed consent, and the study protocol was approved by the Institutional Review Board of Samsung Medical Center (SMC 2021-01-165).

### Baseline characteristics of the study population and the outbreak situation during the study period

**FIG 1:**
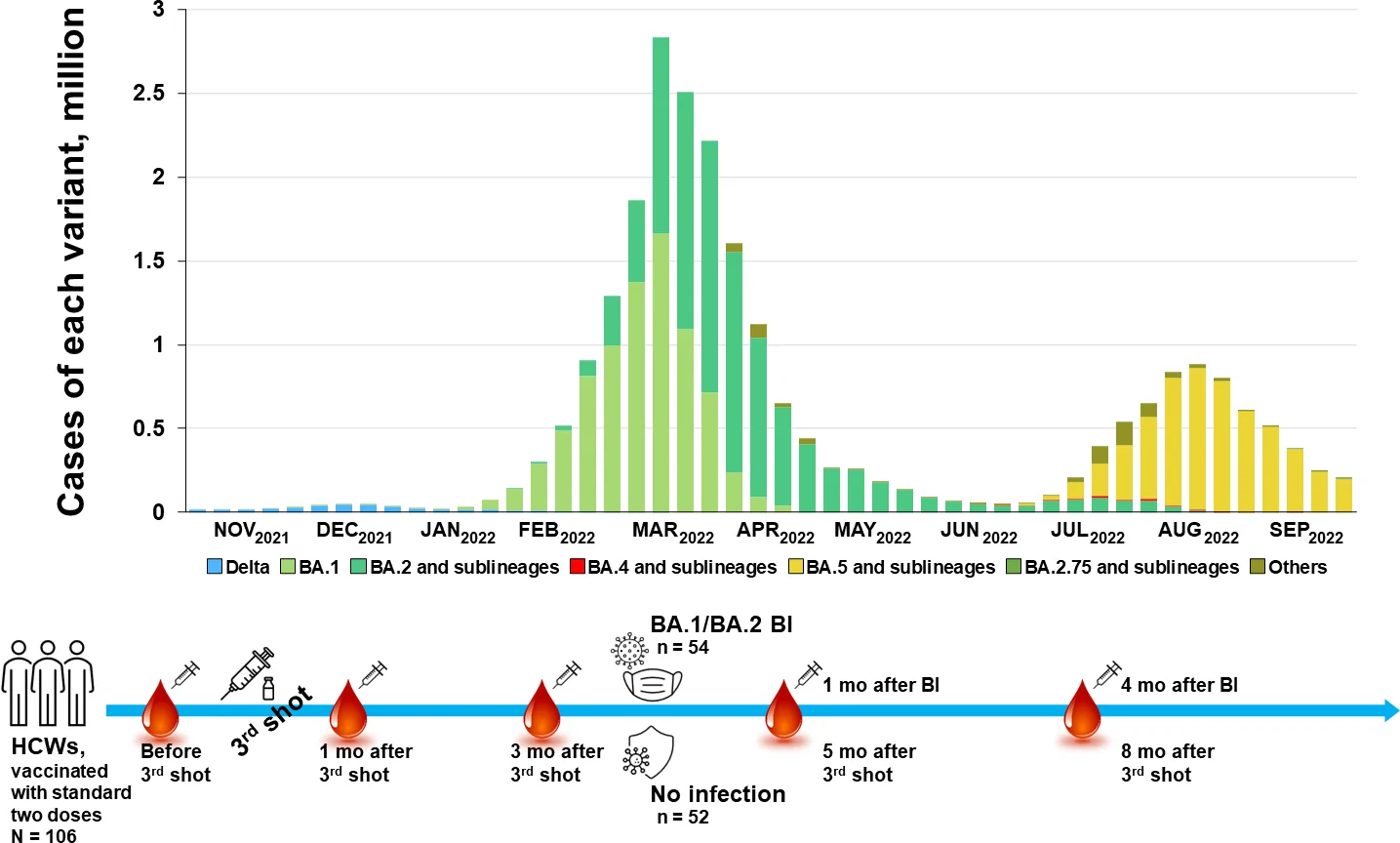
Schematic view of sampling points for the HCW vaccinee cohort according to the omicron-dominated outbreak situation in South Korea, 2022. A total of 106 HCWs vaccinated with the third dose of COVID-19 vaccine were followed for 8 months after the third dose, 54 (50.9%) of whom experienced BI during the BA.1/BA.2 outbreak period.

A total of 106 HCWs were included in the present analysis, with 67 HCWs in the BNT-BNT group and 39 in the ChAd-BNT group (Table 1). Six HCWs were excluded from the total HCW vaccinee cohort of our center (15) because four did not receive a third dose and two experienced COVID-19 before the third vaccination. The average age of the HCWs was 35.3 years, and participants were mostly female (77.4%). The mean body mass index of the HCWs was 22.2 kg/m^2^, and a mere 8.5% of them had comorbidities, the majority of which were mild and well-controlled. The titers of Sab, WT PRNT, Delta PRNT, and BA.1 PRNT significantly increased after the third dose (all *P* < 0.001). Of the HCWs evaluated, 54 (50.9%, 31 in the BNT-BNT group and 23 in the ChAd-BNT group) experienced BI, while 52 did not (49.1%, 31 in the BNT-BNT group and 16 in the ChAd-BNT group). Among 54 HCWs who experienced BI, most of them (*n* = 50, 92.6%) were diagnosed with positive rapid antigen or RT-PCR tests (molecular diagnosis). Those with molecular diagnosis accompanied positive seroconversion of Nab (45/50, 90%) and/or rising of Sab with various degrees (5.4-fold increase in average). Only four HCWs (7.4%) were serologically diagnosed. Three of them showed positive seroconversion of Nab with rising Sab titers (a 4.4-fold increase in average), while one showed a 4.7-fold rise of Sab [12,333 to 57,745 binding antibody unit (BAU)/mL] without seroconversion of Nab. There were no significant differences in demographics, underlying disease, type of previous vaccination, reactogenicity after the third vaccination, Sab titer, or PRNT titer between the HCWs with or without BI. The timeline of sampling is illustrated in Fig. 1 according to the domestic outbreak situation. Sampling was conducted at five-time points, and a total of 510 specimens were analyzed. Twenty sample collections were missed due to personal reasons by the participants. Sab testing was performed for all specimens, and a total of 844 PRNT assays were conducted for WT SARS-CoV-2 and circulating variants at each sampling point. The median interval from the third dose to BI was 127.5 days, with an interquartile range (IQR) of 111.8–147.3 days.

**TABLE 1:** Baseline characteristics and immune response after the third dose, presented according to BI during the BA.1/BA.2 outbreak period^*a*^

Variables	Total HCWs^*c*^ (*n* = 106)	HCWs with BA.1/BA.2 BI (*n* = 54)	HCWs without BA.1/BA.2 BI (*n* = 52)	*P* value
Demographics				
Age, year	35.3 ± 9.5	35.5 **±** 9.6	35.0 ± 9.4	0.787
Male sex	24 (22.6)	13 (24.1)	11 (21.2)	0.450
BMI, kg/m^2^	22.2 ± 3.0	22.2 ± 2.8	22.1 ± 3.2	0.804
Underlying disease				
Hypertension	2 (1.9)	1 (1.9)	1 (1.9)	0.979
Dyslipidemia	1 (0.9)	0 (0.0)	1 (1.9)	0.306
Arrythmia	1 (0.9)	0 (0.0)	1 (1.9)	0.306
Cancer, treated	2 (1.9)	1 (1.9)	1 (1.9)	0.979
Grave’s disease	2 (1.9)	0 (0.0)	2 (3.8)	0.146
Rheumatoid disease	1 (0.9)	0 (0.0)	1 (1.9)	0.306
Vaccination status				
BNT-BNT/ChAd-BNT	67 (63.2)/39 (36.8)	31 (57.4)/23 (42.6)	36 (69.2)/16 (30.8)	0.144
Reactogenicity after third dose	18.0 ± 16.9	17.1 ± 17.6	19.0 **±** 16.2	0.564
Anti-spike antibody, BAU/mL				
Before third dose	1,045.0 ± 2,264.0	1,291.0 ± 3,136.0	789.4 ± 445.7	0.256
After third dose	15,172.0 ± 9,220.0	14,867.9 ± 9,048.1	15,488.1 ± 9,472.3	0.731
PRNT, ND_50_^*b*^				
WT, before third dose	473.4 ± 525.6	473.2 ± 455.2	473.6 ± 643.7	0.999
WT, after third dose	3,331 ± 2,046	3,390.3 ± 1,577.6	3,250.5 ± 2,682.4	0.888
Delta, before third dose	114.2 ± 114.9	118.8 ± 82.8	107.7 ± 155.1	0.842
Delta, after third dose	1,670 ± 1,183	1,770.4 ± 1,168.3	1,531.7 ± 1,269.1	0.677
BA.1, before third dose	43.3 ± 50.7	43.2 ± 64.0	43.4 ± 21.9	0.994
BA.1, after third dose	625.8 ± 475.4	728.3 ± 461.8	484.9 ± 487.1	0.283

^*a*^ Data are expressed as the number (%) of patients or the mean ± standard deviation.

^*b*^ PRNT for these time points was tested in 11 HCWs with BI and 8 HCWs without BI.

^*c*^ BMI, body mass index; BNT, BNT162b2 vaccine; ChAd, ChAdOx1 vaccine.

### Laboratory procedures

For semiquantitative measurement of Sab, an Elecsys Anti-SARS-CoV-2 S kit (Roche Diagnostics, Basel, Switzerland) was utilized (18, 19). This kit uses an electrochemiluminescence immunoassay method and cobas e analyzers. The positive cut-off value is a Sab concentration of 0.8 U/mL, and the linear range is 0.4–250 U/mL. Automated dilution was performed for up to a 1:50 dilution using the analyzer, and additional manual dilutions of up to 1:200 were applied for the saturated specimens. A linear titer-correlation of the Elecsys Anti-SARS-CoV-2 S kit and PRNT was presented in previous publications (18, 19). To standardize binding assay results to the BAUs recommended by the World Health Organization, a correction factor of 0.972 provided by the manufacturer was multiplied by the result (6, 20, 21). To assess the neutralizing activity against WT SARS-CoV-2 and circulating variants, we conducted PRNT on selected specimens (17). PRNT against WT SARS-CoV-2 (NCCP No. 43326) was included as a reference for each test. PRNT against delta (NCCP No. 43390) and BA.1 (NCCP No. 43408) variants was conducted for the specimens collected before (*n* = 19), 1 month after (*n* = 19), and 3 months after the third dose (*n* = 39). PRNT against BA.1, BA.2 (NCCP No. 43412), BA.4 (NCCP No. 43425), and BA.5 (NCCP No. 43426) variants was tested in specimens collected 5 months after the third vaccination (for those who did not experience BI, *n* = 37) or 1 month after BA.1/BA.2 BI (*n* = 40). Specimens collected 8 months after the third dose (*n* = 37) or 4 months after BI (*n* = 39) underwent PRNT against BA.1 and BA.5 variants. PRNT was performed as previously described (17), and the 50% neutralization dose (ND_50_) was calculated using the Karber formula (22). PRNT ND_50_ over 20 was considered positive according to the standard operating procedure of the Korea Disease Control and Prevention Agency, whereas WT PRNT ND_50_ of 118.25 was estimated as a 50% protective value in a previous publication (16).

### Statistical analysis

To compare baseline characteristics and laboratory test results, Student’s *t*-test or Mann-Whitney *U* test was used for continuous variables, and chi-squared or Fisher exact tests were used for categorical variables. A linear regression model was utilized to assess the correlations between antibody titers and timeline, as well as between Sab and PRNT titers. For the interpretation of the correlation coefficient, *R* ^2^ ≥0.7 was considered a strong correlation, *R* ^2^ ≥0.4 a moderate correlation, and *R* ^2^ <0.4 a weak correlation (23). Antibody titers, including Sab and PRNT ND_50_, were evaluated on the log_10_ scale, and correlation equations derived from the linear regression model were used to estimate group immunity. All *P* values were two-tailed, and values <0.05 were considered statistically significant. GraphPad Prism version 8.0 (GraphPad Software, San Diego, CA, USA) was used for the analysis and graph plotting of the results.

## RESULTS

### Sab, WT PRNT, and BA.1 PRNT titers according to the vaccination and BA.1/BA.2 BI

**FIG 2:**
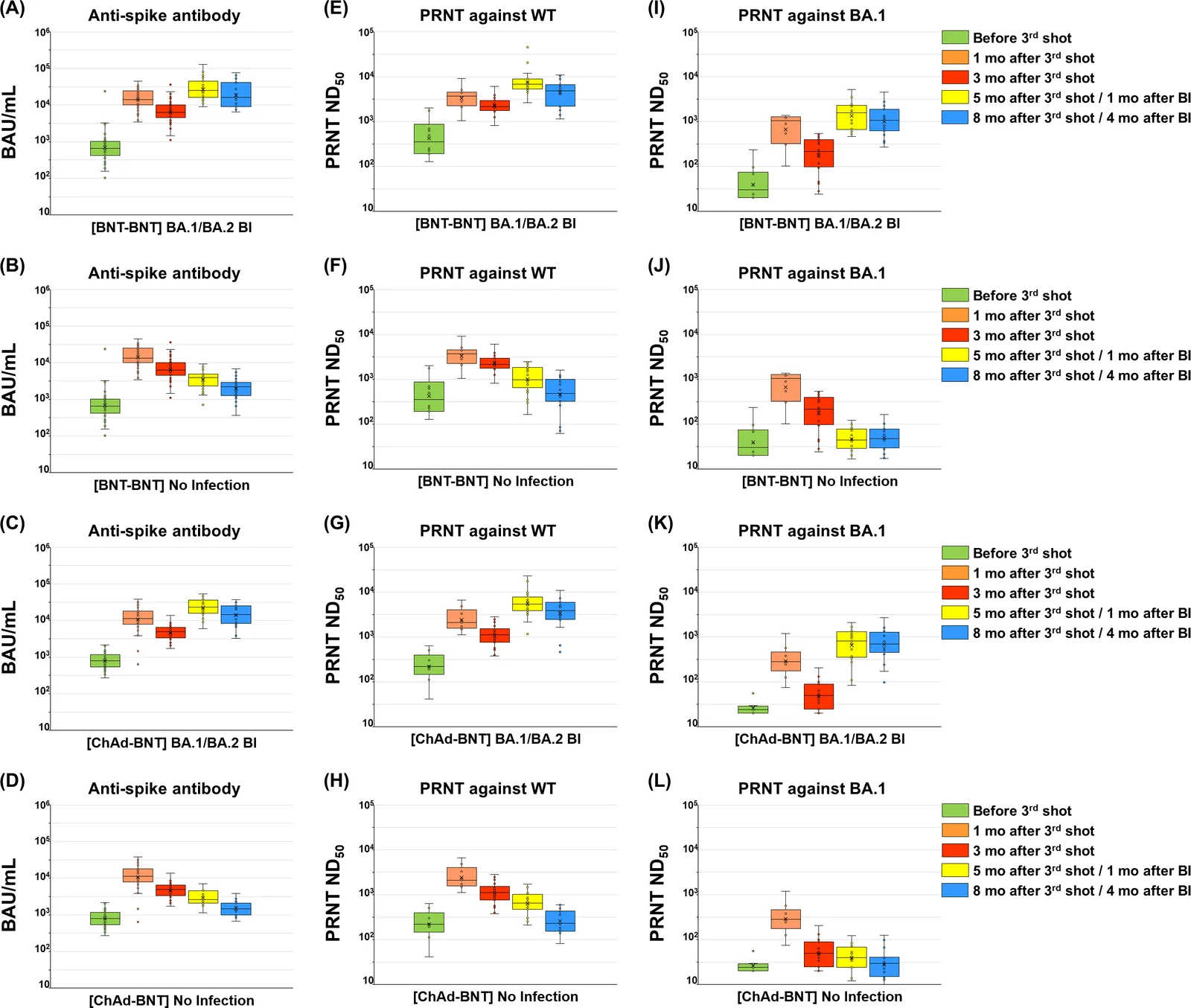
Measured values of Sab, WT PRNT, and BA.1 PRNT titers of each sampling point according to vaccination group and BA.1/BA.2 BI. Sab titers are presented as BNT-BNT vaccination group with (**A**) or without (**B**) BA.1/BA.2 BI and ChAd-BNT vaccination group with (**C**) or without (**D**) BI, respectively. In the same manner, WT PRNT titers of the BNT-BNT vaccination group with (**E**) or without (**F**) BI, WT PRNT titers of the ChAd-BNT vaccination group with (**G**) or without (**H**) BI, BA.1 PRNT titers of the BNT-BNT vaccination group with (**I**) or without (**J**) BI, and BA.1 PRNT titers of the ChAd-BNT vaccination group with (**K**) or without (**L**) BI are presented, respectively. Values measured before, 1 month after, and 3 months after the third dose are shared for each graph, irrespective of BI. The BNT-BNT group and the ChAd-BNT group were statistically not different in age (34.2 ± 9.3 vs 37.0 ± 9.7, *P* = 0.143), sex distribution (female 28.4% vs 12.8%, *P* = 0.092), and underlying diseases (all *P* > 0.05). The positive cut-off values are 0.8 U/ml (0.78 BAU/mL) and 20 for the Sab and PRNT assays, respectively. NT, BNT162b2 vaccine; ChAd, ChAdOx1 vaccine.

Since the Sab test, WT PRNT, and BA.1 PRNT were performed consistently at each sampling point (Fig. 2) and categorized by primary vaccination schedules and BA.1/BA.2 BI, before the third dose, Sab titers of the BNT-BNT group and the ChAd-BNT group were 1,132.0 ± 2,831 and 894.9 ± 447.2 BAU/mL, respectively (*P* = 0.605; Fig. 2A through D). One month after the third dose, both groups showed a robust increase in Sab titer, with a significantly higher increase in the BNT-BNT group (16,811.0 ± 9,487.0 BAU/mL) than in the ChAd-BNT group (13,125.0 ± 7,846.0 BAU/mL, *P* = 0.045). The increase after the third dose was also larger in the BNT-BNT group (25.5 ± 14.7-fold increase) than in the ChAd-BNT group (16.1 ± 7.40-fold increase) (*P* < 0.001). After the third dose, the Sab titers waned gradually in both groups without experience of BI, though those in the BNT-BNT group remained higher than those in the ChAd-BNT group (at 3 months, 8,053 ± 5,879 vs 5,094 ± 2,424 BAU/mL, *P* = 0.0046; at 5 months, 5,524 ± 3,237 vs 3,207 ± 1,579 BAU/mL, *P* = 0.004; at 8 months, 4,464 ± 3,511 vs 1,644 ± 820.4 BAU/mL, *P* = 0.002). When comparing the individual waning slopes of Sab titers as a log_10_ scale, they were not different between the two groups (−0.109 ± 0.032 log_10_BAU/mL/month in the BNT-BNT group and −0.114 ± 0.048 log_10_BAU/mL/month in the ChAd-BNT group, *P* = 0.681). After BI, Sab titers robustly increased to 33,253 ± 25,463 (6.2 ± 3.0-fold, BNT-BNT group) and 25,435 ± 13,000 BAU/mL (5.2 ± 3.5-fold, ChAd-BNT group), without significant differences between the two groups (*P* = 0.161).

WT PRNT exhibited a similar trend but differences between the groups during the waning period (Fig. 2E through H). Before the third dose, the WT PRNT ND_50_ of the BNT-BNT group and the ChAd-BNT group were 650.5 ± 669.2 and 276.6 ± 186.8, respectively (*P* = 0.125). One month after the third dose, both groups showed a robust increase without a significant difference between the two groups (3,826.0 ± 2,200.0, 9.4 ± 5.4-fold in the BNT-BNT group and 2,781.0 ± 1,824.0, 17.2 ± 19.3-fold in the ChAd-BNT group, *P* = 0.279 and 0.237). After the third vaccination, WT PRNT titer decreased continuously in both groups without BI, being still higher in the BNT-BNT group than in the ChAd-BNT group (at 3 months, 2,456.0 ± 1,181.0 vs 1,224.0 ± 684.0, *P* < 0.001; at 5 months, 1,217.0 ± 697.5 vs 738.5 ± 429.2, *P* = 0.021; at 8 months, 640.7 ± 439.0 vs 300.3 ± 167.0, *P* = 0.006). The waning slope as a log scale was not significantly different between the two groups (−0.111 ± 0.067 log_10_PRNT ND_50_/month in the BNT-BNT group and −0.113 ± 0.057 log_10_PRNT ND_50_/month in the ChAd-BNT group, *P* = 0.937). After BI, WT PRNT increased robustly to 9,399.0 ± 9,159.0 in the BNT-BNT group and 7,238.0 ± 4,968.0 in the ChAd-BNT group (*P* = 0.370). The fold increase was not different between the two groups (4.6 ± 4.8-fold vs 7.2 ± 5.0-fold, respectively, *P* = 0.112).

BA.1 PRNT, which were much lower than WT PRNT, also exhibited a similar trend but showed differences between the vaccination groups during the waning period (Fig. 2I through L). Before the third dose, BA.1 PRNT ND_50_ of the BNT-BNT group and the ChAd-BNT group were 58.1 ± 67.2 and 26.7 ± 11.2, respectively (*P* = 0.189). One month after the third dose, both groups showed a robust increase in BA.1 PRNT titer, with a significantly higher titer in the BNT-BNT group (849.0 ± 484.6) than in the ChAd-BNT group (377.9 ± 336.0, *P* = 0.026). The fold-increase of BA.1 PRNT titer after the third dose was not significantly different between the two groups (28.2 ± 26.6-fold vs 15.4 ± 14.4-fold, respectively, *P* = 0.216). Three months after the third dose, BA.1 PRNT titer waned in both groups but remained higher in the BNT-BNT group (248.9 ± 171.6) than in the ChAd-BNT group (59.7 ± 47.1, *P* < 0.001). Among HCWs without BI, BA.1 PRNT titers decreased continuously in both groups, showing similarly low titers at 5 months after the third dose (54.2 ± 29.9 vs 47.2 ± 30.7, *P* = 0.491) and at 8 months after the third vaccination (56.7 ± 33.6 vs 37.1 ± 32.7, *P* = 0.083). The waning slope on the log scale was not significantly different between the two groups (−0.051 ± 0.083 log_10_PRNT ND_50_/month in the BNT-BNT group and −0.038 ± 0.070 log_10_PRNT ND_50_/month in the ChAd-BNT group, *P* = 0.608). After BI, BA.1 PRNT increased robustly and was higher in the BNT-BNT group (1,676.0 ± 1,212.0) than in the ChAd-BNT group (861.3 ± 579.1, *P* = 0.008). The fold increase was not different between the two groups (14.7 ± 18.1-fold vs 22.0 ± 17.7-fold, respectively, *P* = 0.219).

### Cross-reactive neutralizing activity against circulating variants

**FIG 3:**
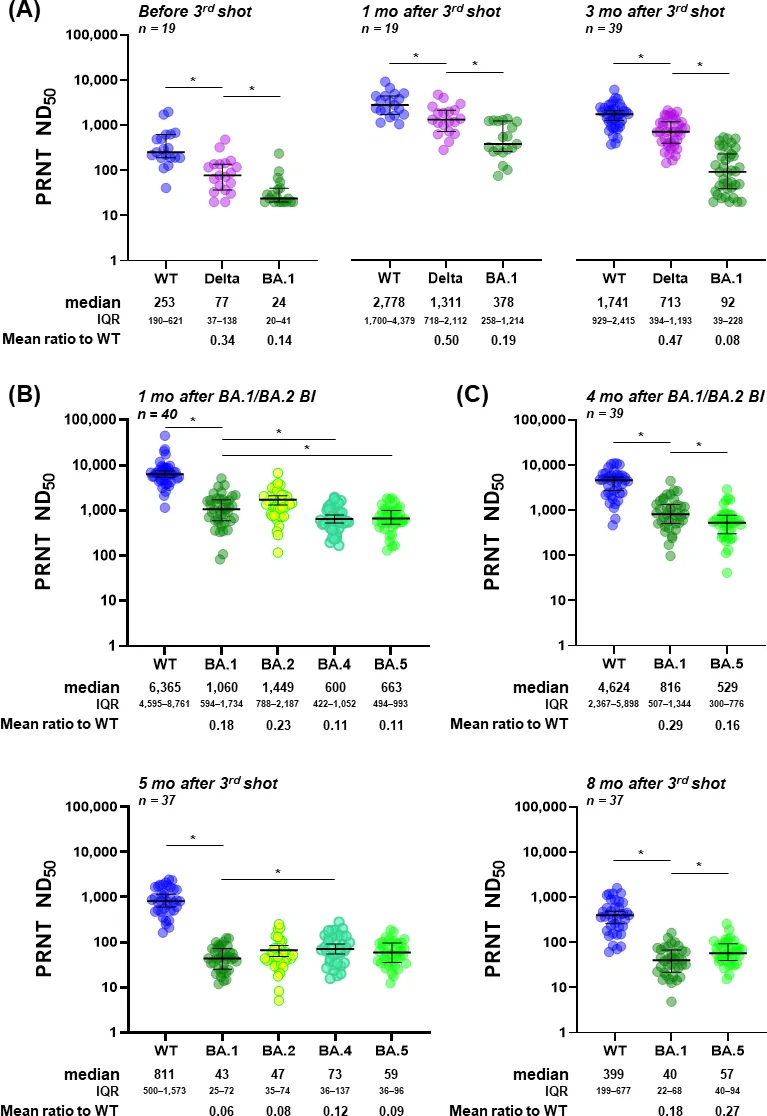
PRNT titers against SARS-CoV-2 variants circulating at each sampling point. PRNT against WT, delta, and BA.1 was tested before, 1 month after, and 3 months after the third dose (**A**). At 5 months after the third vaccination (for those who did not experience BI) or 1 month after BA.1/BA.2 BI, PRNT against WT, BA.1, BA.2, BA.4, and BA.5 were tested (**B**). At the sampling point of 8 months after the third dose or 4 months after BI, PRNT against WT, BA.1, and BA.5 was conducted. The ratio to WT was calculated from individual titers, and the mean value was presented in the figure. The positive cut-off value is 20 for the PRNT assay.

Cross-reactive neutralizing activity against circulating variants is presented in Fig. 3. PRNT was conducted against WT, delta, and BA.1 in samples collected before, 1 month after, and 3 months after the third dose (Fig. 3A). At each sampling point, the ND_50_ of WT PRNT was highest, followed by those of delta PRNT and BA.1 PRNT (all *P* < 0.05). One month after the third dose, delta PRNT showed a robust increase, and the PRNT ratio to WT increased significantly (from 0.34 ± 0.23 to 0.50 ± 0.19, *P* = 0.026). Three months after the third dose, the delta PRNT waned along with the WT PRNT, and the PRNT ratio to WT did not differ significantly (0.47 ± 0.18, *P* = 0.588). BA.1 PRNT also increased significantly 1 month after the third dose, but the increase in PRNT ratio to WT was not statistically significant (from 0.14 ± 0.12 to 0.19 ± 0.15, *P* = 0.232). Three months after the third dose, BA.1 PRNT decreased more drastically than WT PRNT, and the PRNT ratio to WT was lower than at previous point (0.08 ± 0.06, *P* < 0.001).

At 1 month after BA.1/BA.2 BI or 5 months after the third dose without BI, PRNT was conducted against WT and globally circulating omicron subvariants, including BA.1, BA.2, BA.4, and BA.5 (Fig. 3B). One month after BA.1/BA.2 BI, WT PRNT increased more than it did 1 month after the third dose (*P* = 0.008). PRNT against omicron subvariants also increased. The ND_50_ between BA.1 and BA.2 PRNT and that between BA.4 and BA.5 PRNT were statistically not different, while the ND_50_ of BA.1 or BA.2 PRNT was significantly higher than that of BA.4 or BA.5 PRNT (all *P* < 0.05). The PRNT ratio to WT of BA.1 (0.18 ± 0.10) and BA.2 (0.23 ± 0.10) was similar to that of BA.1 1 month after the third dose. The PRNT ratios to WT of BA.4 (0.11 ± 0.06) and BA.5 (0.11 ± 0.05) were significantly lower than those of BA.1 1 month after the third dose but higher than those of BA.1 3 months after the third vaccination (all *P* < 0.05). Five months after the third dose without BI, the PRNT ND_50_ of WT and omicron subvariants further decreased. BA.1 PRNT ND_50_ was not statistically different from that measured before the third dose. Although BA.4 PRNT was statistically higher than BA.1 PRNT, all PRNT titers of omicron subvariants remained low, with median values between the positive cut-off value of 20 and the estimated 50% protective value of 118.25 (16).

At 4 months after BA.1/BA.2 BI or 8 months after the third dose without BI, PRNT was conducted against WT, BA.1, and BA.5 (Fig. 3C). BA.2 was omitted at this point as similar cross-reactivity with BA.1 was noticed at the previous sampling point. BA.4, which did not circulate as a main subvariant, was not tested either. Four months after BA.1/BA.2 BI, all PRNT titers decreased compared to the previous point, and WT PRNT remained the highest, followed by BA.1 PRNT and BA.5 PRNT, with a statistically significant difference (all *P* < 0.05). However, the PRNT ratio to WT of BA.1 (0.29 ± 0.29) and BA.5 (0.16 ± 0.12) was increased and significantly higher than that of 1 month after BA.1/BA.2 BI (all *P* < 0.05), suggesting slower waning of BA.1 and BA.5 PRNT than WT PRNT. Eight months after the third dose without BI, WT PRNT titer further decreased, while BA.1 and BA.5 PRNT titers remained at low-positive values, similar to those of 5 months after the third dose.

### Waning kinetics of Sab, WT PRNT, and BA.1 PRNT titers according to BA.1/BA.2 BI

**FIG 4:**
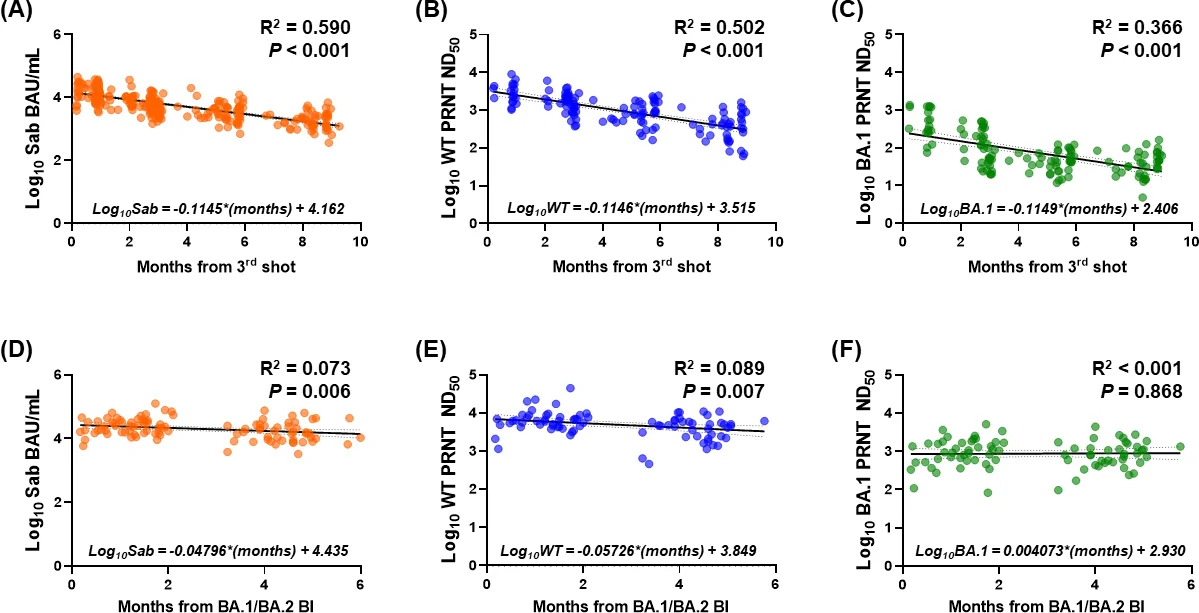
The waning kinetics of Sab and PRNT titers, according to BA.1/BA.2 BI. The kinetics of Sab (**A**), WT PRNT (**B**), and BA.1 PRNT (**C**) among HCWs who did not experience BI during the study period are presented as a timeline from the third dose. For HCWs who experienced BA.1/BA.2 BI, the waning kinetics of Sab (**D**), WT PRNT (**E**), and BA.1 PRNT (**F**) are presented as a timeline from BI. The positive cut-off values are 0.8 U/ml (0.78 BAU/mL) and 20 for the Sab and PRNT assays, respectively.

Waning kinetics of Sab, WT PRNT, and BA.1 PRNT titers after the third dose were obtained using a linear regression model, according to BA.1/BA.2 BI (Fig. 4). After the third dose, log_10_Sab BAU/mL (*R* ^2^ = 0.590) and log_10_WT PRNT ND_50_ (*R* ^2^ = 0.502) exhibited a moderate linear correlation with month, while log_10_BA.1 PRNT ND_50_ (*R* ^2^ = 0.366) showed a significant but weak linear correlation (all *P* < 0.05). The waning slope was similar between Sab (−0.1145), WT PRNT (−0.1146), and BA.1 PRNT (−0.1149). After BA.1/BA.2 BI, a linear correlation was noted between log_10_Sab BAU/mL (*R* ^2^ = 0.073) or log_10_WT PRNT ND_50_ (*R* ^2^ = 0.089) by month (both *P* < 0.05). The slope became very gradual (−0.04796 and −0.05726, respectively), and the correlation of log_10_ antibody titer with month was weak. The waning of BA.1 PRNT was much slower after BA.1/BA.2 BI (with a slope of −0.004073), and the linear correlation with month was not significant in this estimation (*R* ^2^ <0.001, *P* = 0.868). This estimation suggests that the log_10_ values of antibody titers were linearly correlated with the month after the third dose, and the waning became much slower after BA.1/BA.2 BI, especially for BA.1 PRNT titers.

### Correlation equations between Sab and PRNT titers according to BA.1/BA.2 BI

**FIG 5:**
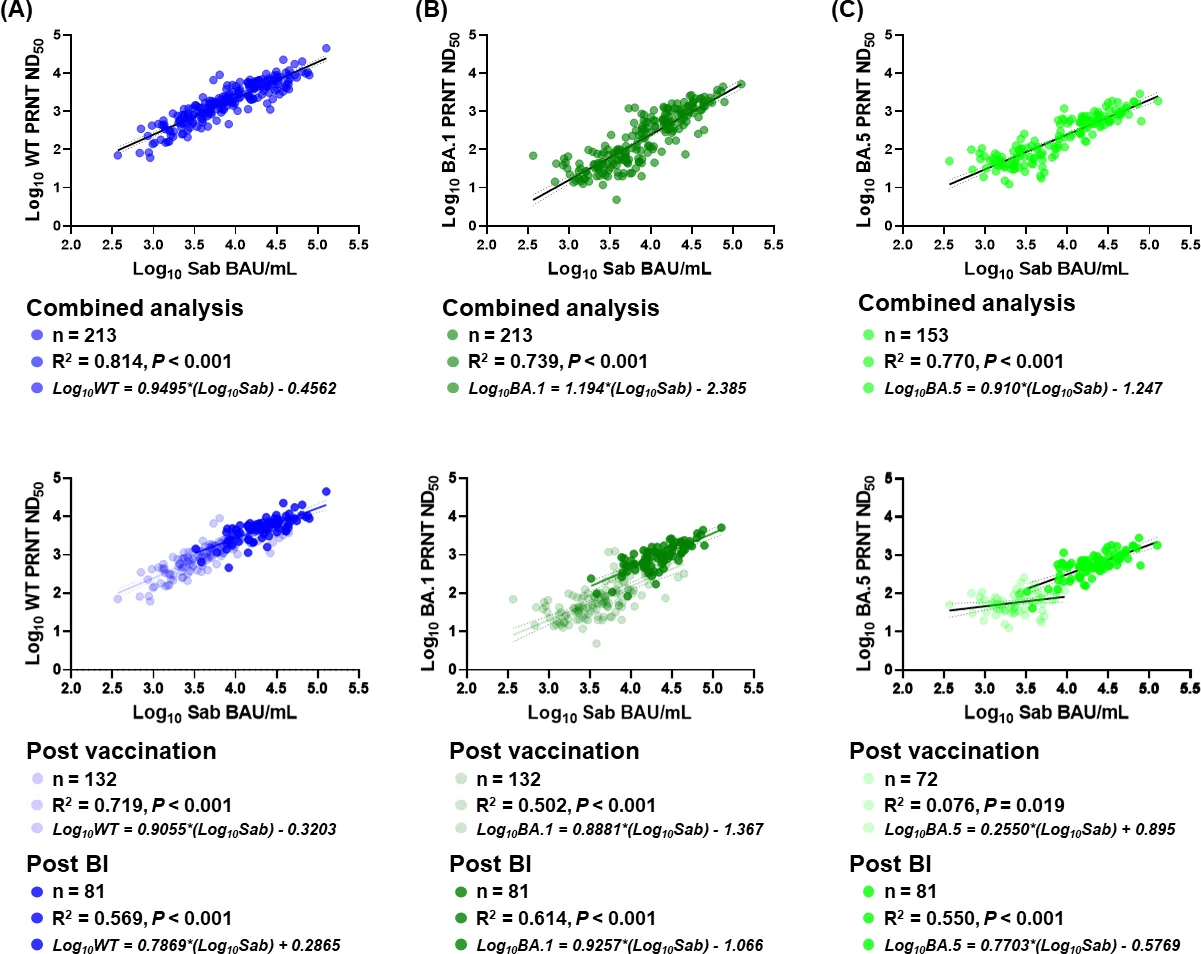
Correlation equations between Sab and PRNT titers. Correlation equations of the log_10_Sab titer with the log_10_ of WT PRNT (**A**), BA.1 PRNT (**B**), and BA.5 PRNT (**C**) titers were deduced. The analysis was performed for total specimens (combined analysis), specimens obtained after BA.1/BA.2 BI (post-BI), and specimens obtained from HCWs who did not experience BI or before BI (post-vaccination), respectively. The positive cut-off values are 0.8 U/mL (0.78 BAU/mL) and 20 for the Sab and PRNT assays, respectively.

The correlation equations were derived from log_10_ values of Sab titer and WT, BA.1, and BA.5 PRNT titers using a linear regression model (Fig. 5). These were primarily conducted using total measured values (combined analysis) and then evaluated separately as post-vaccination without BI and post-BI. In the combined analysis, log_10_WT PRNT ND_50_ (*R* ^2^ = 0.814), log_10_BA.1 PRNT ND_50_ (*R* ^2^ = 0.739), and log_10_BA.5 PRNT ND_50_ (*R* ^2^ = 0.770) showed a strong linear correlation with log_10_Sab BAU/mL (all *P* < 0.001). When the analysis was performed separately according to BA.1/BA.2 BI, a strong correlation between WT PRNT and Sab (*R* ^2^ = 0.719) among post-vaccination specimens became moderate (*R* ^2^ = 0569) among post-BI specimens. In contrast, the *R* ^2^ calculated between BA.1 PRNT and Sab among post-vaccination specimens (0.502) increased to 0.614 in the analysis of post-BI specimens. Such an increase was more apparent in the correlation between BA.5 and Sab, whose *R* ^2^ increased from 0.076 to 0.550. Taken together, the log_10_ values of Sab titers exhibited a linear correlation with the log_10_ values of PRNT titers. The correlation of Sab titers best fits with WT PRNT after the third dose, but the correlation with BA.1 PRNT was much improved after BA.1/BA.2 BI, suggesting that binding antibodies may reflect the neutralizing effect of the circulating variant after BI.

### Estimation of group immunity after the third dose and BA.1/BA.2 BI

**FIG 6:**
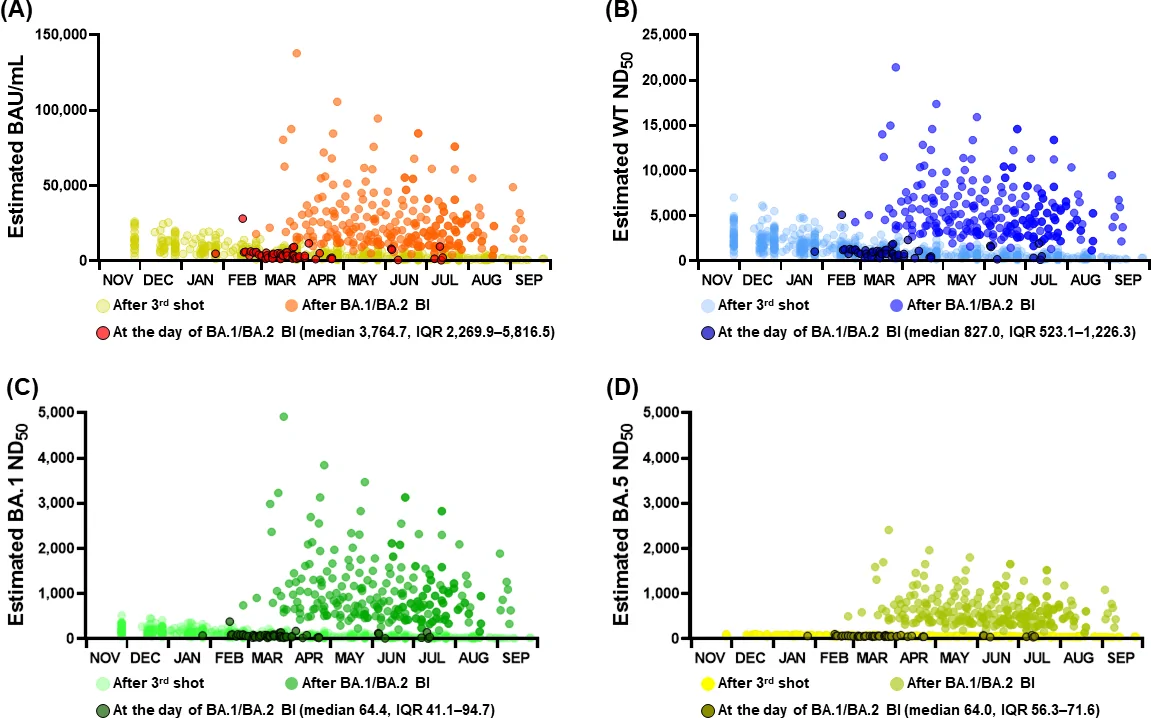
Estimated humoral immune status of the HCW vaccinee cohort changing through BA.1/BA.2 BI. Based on the waning equation of Sab titers and measured values, the monthly Sab titer of each patient was calculated from 1 to 9 months after the third dose, in 1-month interval (**A**). For HCWs who experienced BA.1/BA.2 BI, the monthly Sab titer was calculated for up to 5 months after BI and presented up to September 2022. The calculated Sab titer was converted to WT PRNT (**B**), BA.1 PRNT (**C**), and BA.5 PRNT (**D**) titers, respectively. Estimated values after vaccination were presented in pale colors, while those after BI were depicted in vivid colors. Estimated values on the day of BI were calculated and presented separately with an outlined circle. The positive cut-off values are 0.8 U/mL (0.78 BAU/mL) and 20 for the Sab and PRNT assays, respectively.

**FIG 7:**
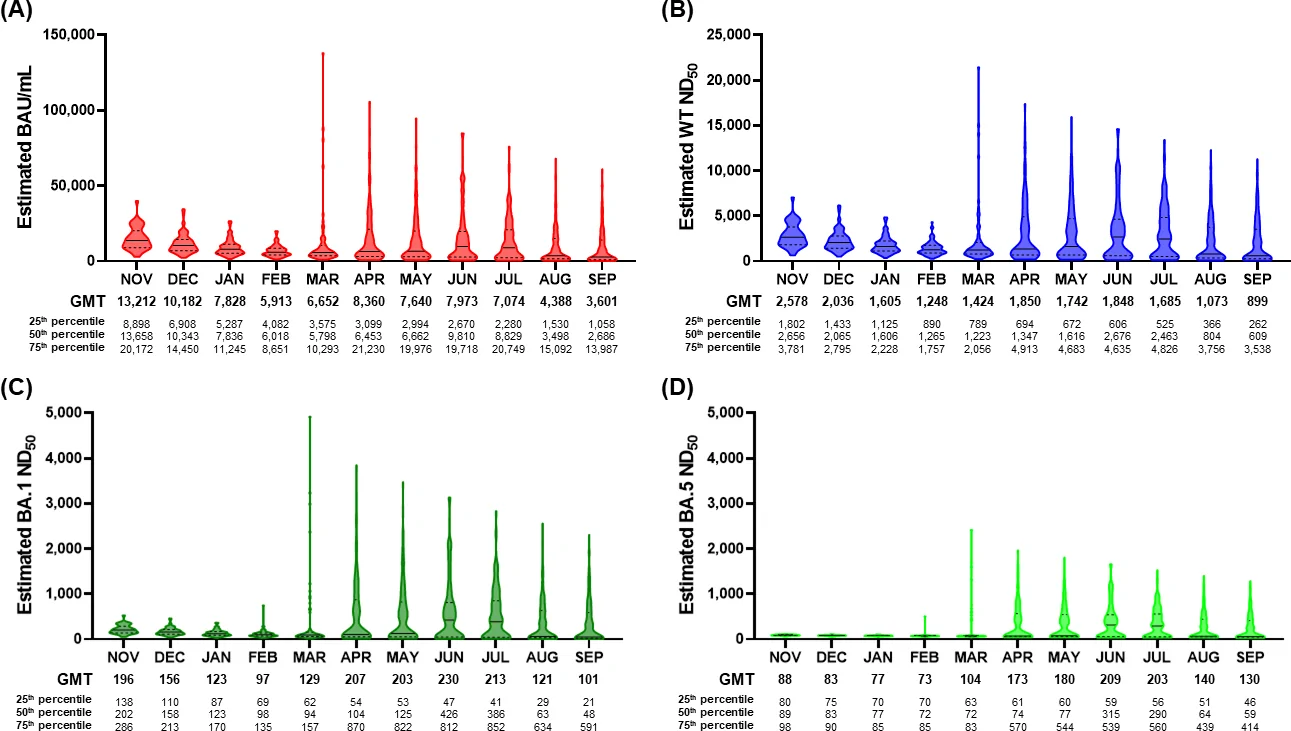
Estimated monthly values of Sab, WT PRNT, BA.1 PRNT, and BA.5 PRNT for an 11-month period after the third dose, reflecting BA.1/BA.2 BI. Estimated monthly titers of Sab (**A**), WT PRNT (**B**), BA.1 PRNT (**C**), and BA.5 PRNT (**D**) were calculated by September 2022 for each HCW. Individual information about the diagnosis of COVID-19 was collected by September 2022, and no one was newly infected after the end of the BA.1/BA.2 outbreak period (after July 24) during the estimation period. One patient was infected on 16 September, but that was after the last estimation date (14 September, 11 months from the third dose). The positive cut-off values are 0.8 U/mL (0.78 BAU/mL) and 20 for the Sab and PRNT assays, respectively.

Based on the equations between log_10_Sab BAU/mL and month (Fig. 4A and D), Sab titers of individual HCWs were re-calculated from the date of the third dose in 1-month intervals (Fig. 6A). Among the HCWs who experienced BA.1/BA.2 BI, Sab titers at the day of BI were estimated (median 3,764.7, IQR 2,269.9–5,816.5 BAU/mL). After BI, Sab titers were estimated from the date of BI in 1-month intervals. The calculated values were then converted to WT, BA.1, and BA.5 PRNT ND_50_ (Fig. 6B–D), using the equations described in Fig. 5. Although all the estimated WT PRNT ND_50_ at the day of BI (median 827.0, IQR 523.1–1,226.3) were higher than the previously estimated 50% protective level of 118.25 (16), most of the estimated BA.1 PRNT ND_50_ (86.8%) was lower than 118.25 (median 64.4, IQR 41.1–94.7). The estimated BA.5 PRNT ND_50_ after the third dose was much lower than BA.1 PRNT, but the values increased and waned slowly after BA.1/BA.2 BI. To depict the estimated level of group immunity of the HCW vaccinee cohort, values are presented as a violin plot along with the geometric mean titer (GMT) and percentile value (Fig. 7). The estimated GMT of Sab was highest in November 2021, reflecting the effect of the third dose, and waned thereafter. After the surge of the BA.1/BA.2 outbreak in February, the Sab GMT increased from March and peaked in April, reflecting BA.1/BA.2 BI. The changing trend of the estimated WT PRNT titer was similar to that of Sab. The vaccine-induced BA.1 PRNT titer also decreased after the third dose, and the GMT became lower than 118.25 from February 2022, the time point that the BA.1/BA.2 outbreak started (Fig. 1). The peak GMT of BA.1 PRNT was observed in June, after the end of the BA.1/BA.2 wave. The GMT of BA.5 PRNT also increased because of cross-reactive neutralizing activity induced by BA.1/BA.2 BIs, peaking in June and waning thereafter. When the estimated titers at the beginning of the BA.1/BA.2 (GMT 97, February 2022) and BA.5 (GMT 203, July 2022) outbreak waves were compared, the BA.5 PRNT titer of July was significantly higher than the BA.1 PRNT titer of February (*P* < 0.001), resulting in the smaller size of the BA.5 outbreak than the BA.1/BA.2 wave. Taken together, the estimated group immunity correlated with the actual outbreak situations, and estimated PRNT titers against the circulating variants seemed to be associated with outbreak size.

## DISCUSSION

Serologic investigation of SARS-CoV-2 has been conducted widely for various purposes, including serologic diagnosis, estimating levels of herd immunity, and evaluating the vaccine’s immunogenicity (24 – 27), which is essential for establishing future vaccination strategies (6, 28). Real-time measurement of neutralizing activity against the circulating variants would not be feasible because substantial time is required for the isolation of the newly emerging variants and the conduct of neutralizing tests. Nevertheless, it would be important to estimate the effects of vaccination and BI in conjunction with outbreak situations, which can be extrapolated into upcoming outbreaks. A modeling study by Hatzakis et al. suggested that a spline model could depict waning antibody kinetics better than an exponential model (29). Spline models can reflect infection points, therefore being more suitable if slope-changing points are present (30). In the present analysis, we utilized equations of waning antibody titers and Sab-PRNT correlations deduced by a linear regression. We categorized specimens into waning after vaccination and waning after BI and noticed that the waning slope differs between the two groups. Employing respective correlation equations according to the status of vaccination and BI, we could estimate the longitudinal kinetics of a vaccinee cohort, reflecting the overall outbreak situation.

In the present analysis, we depicted the longitudinal kinetics of Sab and PRNT against circulating variants in the vaccinee cohort of HCWs, reflecting group immunity after the third dose of COVID-19 vaccination and BA.1/BA.2 BI during the 2022 COVID-19 outbreak in the Republic of Korea. We adopted the previously calculated 50% protective value of PRNT ND_50_ 118.25, interpreting the measured and estimated PRNT titers in the present analysis (16). Along with WT PRNT, delta PRNT titers were robustly boosted 1 month after the third dose and maintained at about 5.7-fold higher than the 50% protective value 3 months after the third dose. No one experienced BI during the delta-dominated fourth wave in the present cohort. However, the cross-reactivity of vaccine-induced immunity to omicron subvariants was insufficient and waned rapidly, consistent with previous reports (31). The estimated GMT of BA.1 PRNT became lower than the 50% protective value in February, at the beginning of the BA.1/BA.2 outbreak wave. About 63.5% of HCWs had lower BA.1 PRNT titers than the 50% protective value, and about 50.9% of the present cohort experienced BI during this period, whereas there was no difference in antibody titers between HCWs who experienced BI and those who did not. These results suggest that most individuals did not have sufficient protective immunity against the omicron variant and there were non-pharmaceutical measures, such as exposure burden to SARS-CoV-2, compliance with mask-wearing, or avoiding crowded places during the BA.1/BA.2 outbreak period. Although WT PRNT titer remained highest throughout the study period, BA.1 PRNT increased by a higher degree (10.5-fold) than WT PRNT (4.2-fold) after BI. Cross-reactive neutralizing activity with BA.2 was noted with similar titers as in previous reports (31 – 33). PRNT against BA.4/BA.5 exhibited effective neutralization, but the titers were lower than those against BA.1/BA.2, likely due to the additional mutations in the receptor binding domain of BA.4/BA.5 (33). The waning slope of BA.1 PRNT after BA.1/BA.2 BI was much less steep than that against WT PRNT, and both BA.1 and BA.5 PRNT ratios to WT PRNT increased 4 months after BI. HCWs in the BI group were first exposed to a heterologous antigen by BA.1/BA.2 infection after a vaccination series based on the original strain. Although numerical PRNT titers against WT were higher than those against omicron variants even after BI, fold changes of PRNT titers and slow waning slopes suggest that omicron BI preferentially boosted and sustained neutralizing activity against BA.1 rather than WT. Taken together, these findings suggest that boosted immunity by BI may overcome immune imprinting by the vaccination series based on WT SARS-CoV-2.

This study has several limitations. First, the number of specimens and target SARS-CoV-2 strains varied according to sampling points. As the HCW vaccinee cohort study has been conducted according to the National Vaccination Vaccine Introduction Policy, the PRNT results of each point need to be used as reference data for the decision of healthcare policy, and the target SARS-CoV-2 strains need to reflect the circulating strain at each sampling point (15). In the initial phase of the cohort, we conducted WT PRNT for around 100 specimens from three vaccination groups, but the number of specimens unavoidably decreased to conduct PRNT for several variants simultaneously (17). Nevertheless, we increased target specimens after BI and could focus on the investigation of the overall effects of serial vaccination and BI on group immunity. Second, BI was confirmed by RT-PCR, antigen assay, or serologic analysis, but sequencing data to determine the SARS-CoV-2 variants that caused the infections were not identified. However, in a previous study that was conducted during the BA.1/BA.2 outbreak period, we performed variant sequencing and identified that all the included patients were infected with the BA.1/BA.2 variant (34). As the outbreak waves were dominated by BA.1 and BA.2 and there was substantial cross-reactivity between BA.1 and BA.2 PRNT, the present study represents the serologic effect of BI that occurred during the BA.1/BA.2 outbreak wave. Meanwhile, the seventh domestic outbreak wave followed in November 2022 with a lower peak and smaller rates of increase and decrease and ended in February 2023 (35). In this period, a transition of dominant circulating strain occurred from BA.5 to BN.1, and the bivalent vaccination was introduced. Although the present analysis suggests that antigenic imprinting could be overcome through BI, the effects of bivalent vaccination need to be evaluated separately. Since bivalent vaccines maintain the spike protein sequence of WT SARS-CoV-2, they may strengthen the antigenic imprinting of the WT virus to some extent. Previous publications exhibited the additional protective effect of bivalent boosting over monovalent boosting (36, 37), while the difference in neutralizing antibodies against BA.4/BA.5 was not remarkable between the two vaccine types (38, 39). Follow-up serologic analysis needs to be conducted to assess the herd immunity of our community, and the mixed effect of BI and bivalent vaccination needs to be considered more meticulously.

In conclusion, BA.1/BA.2 BI after the third dose elicited robust and broad neutralizing activity, preferentially maintaining cross-neutralizing longevity against BA.1 and BA.5. The estimated kinetics on the magnitude, breadth, and durability of neutralizing activity provide an overview of group immunity augmented through the third vaccination and BA.1/BA.2 BI, correlating with the actual outbreak situation. Further analysis of the following outbreak waves and bivalent vaccination may support the establishment of public health strategies against COVID-19 endemicity.

## Data Availability

All data in this study are contained within the manuscript.
